# Studying SARS-CoV-2 vaccine hesitancy among health professionals in Tunisia

**DOI:** 10.1186/s12913-022-07803-y

**Published:** 2022-04-12

**Authors:** Nawel Zammit, Amani El Gueder, Aïcha Brahem, Imen Ayouni, Rim Ghammam, Sihem Ben Fredj, Chaima Sridi, Asma Chouchene, Houda Kalboussi, Olfa El Maalel, Souhaeil Chatti, Jihene Maatoug, Hassen Ghannem, Néjib Mrizak

**Affiliations:** 1 Department of Epidemiology (LR19SP03), Faculty of Medicine of Sousse, Farhat Hached University Hospital, University of Sousse, Sousse, Tunisia; 2 Department of Occupational Medicine and Environmental Pathology (LR19SP03), Faculty of Medicine of Sousse, Farhat Hached University Hospital, University of Sousse, Sousse, Tunisia; 3 Faculty of Dental Medicine of Monastir, University of Monastir, Monastir, Tunisia

**Keywords:** COVID-19 Vaccines, Health Personnel, Attitude of Health Personnel, Tunisia

## Abstract

**Background:**

People's lives were seriously affected by the emergence and the spread of the COVID-19 disease. Several vaccines were developed in record time to overcome this pandemic. However, putting an end to this public health problem requires substantial vaccination coverage rate. This latter depends on the acceptance of these vaccines especially by health professionals; the leaders of the current war against COVID-19. In fact, they have a central role in promoting vaccination against the SARS-CoV-2. In the developed countries, hesitancy rates towards these vaccines among health professionals vary from 4.3% to 72%. In the developing countries, few studies focused on this issue.

**Objective:**

To estimate the prevalence and the predictors of SARS-CoV-2 vaccine hesitancy among the Tunisian health professionals.

**Methods:**

A cross-sectional study was led online between the 7th and the 21th of January 2021 among Tunisian health professionals. At least 460 participants were required. Snowball sampling method served to recruit participants. Data were collected using a pre-established and pre-tested questionnaire recorded in a free Google form. The link of the questionnaire was disseminated online to be self-administered anonymously to the participants. The generated online Google Sheet was uploaded and exported to SPSS software for analysis.

**Results:**

Of the 546 responses, 493 were retained. The mean age of participants was 37.4 (± 9.5) years. Females represented 70.2% of participants. Social media represented the most frequently used source of information about COVID-19. The prevalence of SARS-CoV-2 vaccine hesitancy among participants was 51.9% (95% CI_:_ 47.5–56.3)). Female sex, working far from the capital and having concerns about the vaccines components predicted more hesitancy among participants. In contrast, the use of the national COVID-19 information website predicted less hesitancy among them.

**Conclusions:**

The current Tunisian communication plan about COVID-19 vaccines must be reinforced. Social media represent a cost effective communication channel that can serve to reassure Tunisian health professionals regarding the safety of COVID-19 vaccines. Special interest should be paid to females, paramedical professionals and those working far from the capital.

## Background

The new corona virus disease (COVID-19) has drastically altered people's lives worldwide [1]. One year after declaring this new disease as a Public Health emergency, the number of deaths caused by this new disease exceeded two millions [2]. Measures such as lockdowns, social distancing, travel restrictions and mandatory mask wearing limited people’s freedom, triggered psychological issues, reduced the income of the disadvantaged groups and worsened the existing social and health inequalities [1, 3]. Until now, there is no specific treatment for this new disease. Nonetheless, several vaccines were developed in record time and were authorized for emergency use in a wide range of countries [4]. Indeed, speed vaccination of people is required not only to cut the spread of the severe acute respiratory syndrome corona virus 2 (SARS-CoV-2) but also to avoid the emergence and the spread of new variants threatening the efficacy of these vaccines [5]. However, tackling this pandemic requires substantial vaccination coverage rate [6]. This latter is conditioned by the acceptance of these vaccines especially by health professionals [7, 8]. In fact, being at the frontline of the war against the SARS-CoV-2 without immunization, expose them to a supplementary risk of contracting the SARS-CoV-2 and may increase the spread of COVID-19 among the users of healthcare facilities [8]. Indeed, according to a systematic review published in November 2020, the seroprevalence of SARS-CoV-2 antibodies among health professionals did not exceed 45.3% with an overall seroprevalence of 8.7% [9]. Otherwise, health professionals have a central role in promoting vaccination against the SARS-CoV-2 as they are considered trustworthy by the general public [10, 11]. Accordingly, the delay in accepting the SARS-CoV-2 vaccines among health professionals would slow down the attainment of the required vaccination coverage. The majority of studies led about hesitancy towards COVID-19 vaccination concerned the general population while fewer studies have focused on health professionals [7]. A scoping review, led mainly in developed countries, reported that the rates of hesitancy towards COVID-19 vaccination in healthcare workers vary from 4.3% to 72% [12]. Reasons behind the hesitancy towards the SARS-CoV-2 vaccination are also varied [13–16]. North Africa is among the suggested regions where more studies should be led in order to address the scope of COVID-19 vaccine hesitancy [7]. In Tunisia, a North African country, the national vaccination program contributed with the concomitant demographic, social, and economic transitions in accelerating the epidemiological transition [17–19]. Thanks to mandatory vaccination, this program succeeded in reducing the incidence rate of several communicable diseases such as tuberculosis, diphtheria, tetanus, pertussis, rubella, mumps and measles with national vaccination coverage rates around 90% [20–22]. The current pandemic represents a new challenge for the Tunisian Health system. During the first wave (March–May, 2020), the prevalence of COVID-19 among health professionals was 14.8% [23]. During the second wave (which reached its peak in 20 January 2021), regarding the delay in obtaining the SARS-CoV-2 vaccines, the Tunisian government announced a lockdown between 14 and 17 January, the closure of schools between 14 and 24 January, the closure of markets and the delay of cultural manifestations between 18 and 24 January [24]. Meanwhile, Tunisian citizens were invited to register on a national website (evax.tn) in order to freely access to the SARS-Cov-2 vaccines when they will be available [25]. This online registration was launched in 15 January 2021 [26].

Estimating the prevalence and the predictors of hesitancy towards the upcoming SARS-CoV-2 vaccines among Tunisian health professionals would document the extent of this issue in Tunisia, identify most hesitant subgroup and guide local and international health authorities and organizations in their strategy to overcome this global health issue. In this context, the objective of the current study was to determine the prevalence and the predictors of SARS-CoV-2 vaccines hesitancy among a sample of Tunisian health professionals.

## Methods

### Study design

A cross-sectional study was led online between the 7^th^and the 21^th^of January 2021 among Tunisian health professionals in order to evaluate their willingness to uptake the SARS-CoV-2 vaccine when it will be available in Tunisia.

### Study population

All Tunisian health professionals represented the target population. The following formula: *n* = [(Z_α/2_) ^2^ × p x (1-p)]/i^2^ was used to calculate the required sample size. A proportion (p) of SARS-CoV-2 vaccine hesitancy of 50%, a precision (i) of 5%, a type one error (α) of 5% and a loss of 20% due to non-eligible participants (not being a Tunisian health professional) were considered which gave a required sample of at least 460 participants.

Given that no updated national list of Tunisian health professionals with contact details was available in Tunisia, random sampling was not possible. Accordingly, the study was led using a snowball sampling. Initially, a multidisciplinary team of two professors, one residency trainee and a Doctor of Dental Medicine disseminated the survey online. They used their own mailing lists to send e-mails and their Facebook profiles to send messages and to post publications in the Facebook groups of Tunisian health professionals (62 Facebook groups were integrated by the investigators). In fact, Facebook is the most popular social media in Tunisia [27]. They targeted Medical Doctors, Pharmacists, Dentists, Health Technicians and Nurses who are graduated or in training. They also recommended to their colleagues in and out of their hospital wards as well as to the participants to disseminate the online survey. In total, almost 2500 e-mails were sent by the investigators with a daily sharing in the Facebook groups of health professionals.

### Data collection

The investigators, based on their experience and a literature review, designed a questionnaire in French language, as it is the academic language in Tunisia. The questionnaire included parts exploring socio-demographic characteristics (age, sex), professional characteristics (field of activity, position, sector, geographic location of health activities and direct contact with hospitalized COVID-19 patients), medical history (chronic disease, allergy, vaccination against influenza during the current season), information about SARS-CoV-2 vaccines, perceptions and attitudes related to the vaccination against SARS-CoV-2. Two other experts (a Public Health Professor and an Occupational Professor) who were familiar with the assessment methods of content validity evaluated the items with respect to appropriate wording, grammar, clarity, understandability and relatedness to Tunisian culture. They were also required to review the items with respect to their relevance. The questionnaire was then pre-tested on a convenience sample of 30 health professionals to assess the acceptability and the understandability of the items. The overall Cronbach’s alpha coefficient value was 0.511. Unclear items and those that were difficult to understand by two or more health professionals were reformulated taking into account their comments and the experts’ opinion. The final version of the questionnaire was recorded in a free Google form with two sections: one for the consent, the other for the entire questionnaire. A question was added at the end of the form to determine whether the participant has previously responded to the same questionnaire in order to identify duplicated responses. To limit missing data, responses to all questions were mandatory before sending the filled form. The link of the questionnaire was disseminated online to be self-administered anonymously to the participants.

### Definition of the hesitancy towards vaccination against SARS-CoV-2

According to the WHO Strategic Advisory Group on Experts (SAGE) on Immunization, vaccine hesitancy refers to a delay in acceptance or refusal of vaccination despite availability of vaccination services [28]. Accordingly, the attitude towards the vaccination against SARS-CoV-2 was measured using the following question: “When the vaccine against SARS-CoV 2 (the virus responsible for the COVID-19 disease) would be available in Tunisia, will you accept to be vaccinated?”. The possible responses were: “Yes, certainly”, “Yes, probably”, “I do not know yet”, “Probably no”, “It depends on the type of the vaccine”, “Certainly no”, “No I have already contracted the COVID-19”, “No it is contra-indicated for me”.

The responses: “Yes probably”, “I do not know yet”, “Probably no” and “It depends on the type of the vaccine” were re-coded to “yes” to indicate the attitude of SARS-CoV-2 vaccine hesitancy. The responses, “Certainly no”, “No I have already contracted the COVID-19”, “No it is contra-indicated for me” were re-coded to “no” to indicate the attitude of refusal. The response: “Yes, certainly” indicated the attitude of acceptance.

### Data analysis

The generated online Google Sheet was uploaded and exported to the Statistical Package for the Social Sciences (SPSS) 10.0 software (IBM Inc, Chicago, IL) for analysis. Responses of participants who were not Tunisian health professionals were deleted. Descriptive statistics were reported as frequencies for categorical variables and as means and standard deviations for quantitative ones. Differences between groups were examined using the Chi-squared (χ^2^) test to compare proportions. When the above test was not applicable, categories of multinomial variables were grouped. Univariate binary logistic regression served to estimate the magnitude of the statistical associations while multivariate logistic regression was performed to identify the predictors of SARS-CoV-2 vaccine hesitancy. The dependent variable was “SARS-CoV-2 vaccine hesitancy”. All factors that were associated with this dependent variable with a significance level less than 25% were included in a multivariable model. Then, a stepwise backward approach was performed. Observations with missing data concerning some variables that were used in the regression models were deleted. Results of the regression models were expressed as odds ratios (ORs) with confidence interval (CI) of 95%. Three models were retained. Model 1 represents the second last model including the eventual confounding covariate “age”. Model 2 represents the last model without the covariate “age”. Model 3 excluded the covariate “Previous SARS-CoV-2 infection” because of an eventual convergence failure. All statistical tests were two-tailed, and *p*-values < 0.05 were considered statistically significant.

### Ethical considerations

The current study was carried out in accordance with the ethical principles of the Declaration of Helsinki. It was approved by the Ethical Committee of Farhat Hached University Hospital (Institutional review board code: 00,008,937). An introducing paragraph explaining the purpose and the conduct of the study preceded the two sections of the Google form. Anonymity of responses was highlighted. Participants had to give consent (using the first section of the Google form) to access to the questionnaire (the second section of the Google form) by clicking on the response “yes” to the following question: “Do you agree to participate in the study?” In case of responding by “No”, the questionnaire was not administered to the user of the link. The response option “I do not want to answer” was added to the questions about sex, age and medical history. Furthermore, to ensure anonymity, first and last names were not collected and e-mail addresses were not collected as well.

## Results

A total of 546 responses to the online questionnaire were obtained with 23 refusal and 523 acceptances. Among those who accepted to participate, 28 were not health professionals and two were not Tunisians. Accordingly, the retained participants accounted for 493.

The mean age of participants was 37.4 (± 9.5) years. Females represented 70.2% of participants. Medical Doctors, Dentists, Pharmacists and Paramedical professionals represented respectively 59.2%, 15.8%, 14.2% and 10.8% of participants. As regards the geographic location of their professional activities, 196 (39.8%), 188 (38.1%) and 105 (21.3%) were working respectively in the North, the Center and the South of Tunisia. Whereas, 82 (16.6%) participants reported daily direct contact with COVID-19 patients. More details about the sociodemographic characteristics are displayed in Table 1.

**Table 1:** Individual characteristics of participants. (*n* = 493)

Socio-demographic characteristics	n	%
** Age**		
< 40 years	326	66.1
≥ 40 years	167	33.9
** Sex**		
Female	346	70.2
Male	131	26.6
** Grade**		
Trainee	102	20.7
Graduated	391	79.3
** Field of activity**		
Medicine	292	59.2
Dentistry	78	15.8
Pharmacy	70	14.2
Paramedical	53	10.8
** Location of activity**		
North of Tunisia	196	39.8
Center of Tunisia	188	38.1
South of Tunisia	105	21.3
** Sector of activity**		
Public	338	68.5
Private	155	31.4
** Frequency of direct contact with COVID-19 inpatients**		
Never	235	47.7
Sometimes	176	35.7
Every day	82	16.6
** History of chronic condition**		
Yes	101	20.5
No	368	74.7
No response	10	2.0
** History of allergy**		
Yes	89	18.1
No	383	77.7
No response	10	2.0
** Vaccination against influenza during the current season**		
Yes	151	30.6
No	342	69.4
** Sources used to be informed about the SARS-CoV-2**		
Social media		
Yes	330	66.9
No	163	33.1
Scientific journals		
Yes	285	57.8
No	208	42.2
Television channels		
Yes	276	56.0
No	217	44.0
Websites of international scientific organizations		
Yes	226	45.8
No	267	54.2
The national website of the Ministry of Health		
Yes	199	40.4
No	294	59.6
The Tunisian website for information about COVID-19: “Covid.tn”		
Yes	191	38.7
No	302	61.3
Radio stations		
Yes	183	27.1
No	310	62.9
The Tunisian website of the Observatory of new and emergent diseases		
Yes	127	25.8
No	366	74.2
Newspapers		
Yes	96	19.5
No	397	80.5
The website of the Pasteur institute of Tunis		
Yes	42	8.5
No	451	91.5
The Tunisian website for health professionals: “SAUVE.tn”		
Yes	39	7.9
No	454	92.1
Other sources		
Yes	15	3.0
No	478	97.0
**Perceptions**		
** The upcoming SARS-CoV-2 vaccines contain harmful components**		
Yes	48	9.7
May be	289	58.6
No	156	31.6
** Lack of information about the SARS-CoV-2 vaccines**	403	81.7
Strongly agree	121	24.5
Agree	282	57.2
Disagree	65	13.2
Strongly disagree	25	5.1
** The risk level of infection by SARS-CoV-2**		
Low	19	3.9
Mild	147	29.8
High	218	44.2
Very high	109	22.1
** The risk level of complications in case of infection by SARS-CoV-2**	105	21.3
Low	136	27.6
Mild	252	51.1
High	81	16.4
Very high	24	4.9
** Intention towards the vaccination against SARS-CoV-2**		
Refusal	62	12.6
Certainly no	32	6.5
No, already contracted the COVID-19	30	6.1
No, because of a medical contra-indication	-	-
Hesitancy	256	51.9
Probably yes	108	21.9
Do not know yet	96	19.5
Probably no	47	9.5
It depends on the type of the vaccine	5	1.0
Acceptance		
Yes, certainly	175	35.5

Focusing on the information sources about SARS-CoV-2, social media were the most consulted by participants followed by scientific journals and the television channels with the frequencies of 66.9%, 57.8% and 56% respectively while 39 (7.9%) participants used the national information website for Tunisian health professionals (SAUVE.tn). The other reported sources of information are detailed in Table 1.

Asking participants about their perceptions revealed that 327 (66.3%) were thinking that they have high or very high risk of SARS-CoV-2 infection (Table 1). Among dentists, this prevalence was 74.4% (Table 2). Nonetheless, 105 (21.3%) were thinking that they risk serious complications in case of infection (Table 1). This prevalence was the lowest among paramedical professionals (Table 2). On the other hand, 337 (68.4%) had concerns about the components of the upcoming vaccines. Otherwise, lack of information about the SARS-CoV-2 vaccines was reported by 403 (81.7%) of participants (Table 1).

**Table 2:** Risks perceptions and intentions towards the SARS-CoV-2 vaccines among participants according to the fields of their activities. (*n* = 493)

	**Medical doctor (*n* = 292)**	**Dentists (*n* = 78)**	**Pharmacists (*n* = 70)**	**Paramedical personnel (*n* = 53)**	***p*-value**
**Perceived risk level of infection by SARS-COV-2**					0.124
High or very high	197(67.5)	58 (74.4)	41(58.6)	31 (58.5)	
Low or mild	95 (32.5)	20 (25.6)	29 (41.4)	22 (41.5)	
**Perceived risk level of complications in case of infection by SARS-CoV-2**					0.395
High or very high	64 (21.9%)	16 (20.5%)	18 (25.7%)	7 (13.2%)	
Low or mild	228 (78.1%)	62 (79.5%)	52 (74.3%)	46 (86.8%)	
**Perception of a lack of information about the vaccination against SARS-CoV-2**					0.194
Agree or strongly agree	235 (80.5)	70 (89.7)	54 (77.1)	44 (83.0)	
Disagree or strongly disagree	57 (19.5)	8 (10.3)	16 (22.9)	9 (17.0)	
**Concerns about the components of the upcoming vaccines**					0.647
Yes	202 (69.2%)	56 (71.8%)	46 (65.7%)	33 (62.3%)	
No	90 (30.8%)	22 (28.2%)	24 (34.3%)	20 (37.7%)	
**Intention towards the SARS-CoV-2 vaccines**					0.103
Refusal	33 (11.3%)	8 (10.3%)	8 (11.4%)	13 (24.5%)	0.051
Hesitancy	150 (51.4%)	41 (52.6%)	36 (51.4%)	29 (54.7%)	0.974
Acceptance	109 (37.3%)	29 (37.2%)	26 (37.1%)	11 (20.8%)	0.131

Of the 493 respondents, 256 (51.9%; 95% CI_:_ 47.5–56.3)) reported SARS-CoV-2 vaccine hesitancy while 62 (12.6%; 95% CI: 9.7–15.5) were sure to refuse it and 175 (35.5%; 95% CI: 31.3–39.7) were sure to accept it when it will be available in Tunisia (Table 1). Refusal was the highest among paramedical professionals (Table 2).

Proportion of health professionals under the age of 40 years was significantly superior (72.3%) among those hesitating to get the vaccine than those not hesitating (59.5%) (*p* = 0.003). Similarly, females represented 74.6% of those who hesitate against 65.4% in those who do not with a p value of 0.047. Concerning the professional activity, working far from the North of Tunisia or in public sector, were significantly associated with more hesitancy towards the SARS-CoV2 vaccination (Table 3). However, having already contracted the COVID-19 was negatively associated with SARS-CoV2 vaccine hesitancy (0.4% among hesitating participants versus 12.2% among those not hesitating (*p* =  < 0.001)).

**Table 3:** Hesitancy towards the SARS-CoV-2 vaccines according to the individual characteristics of participants. (*n* = 493)

(*n* = 493)	Hesitancy towards the SARS-CoV-2 vaccines			OR, 95% CI
	**Yes (*n* = 256)**	**No (*n* = 237)**	**p**	
**Age**			0.003	
≥ 40 years	71 (27.7)	96 (40.5)		1
< 40 years	185(72.3)	141 (59.5)		1.8 [1.2–2.6]
**Sex**			0.047	
Male	59 (23.0)	72 (30.4)		1
Female	191(74.6)	155 (65.4)		1.5 [1.1–2.2]
**Grade**			0.263	
Graduated	198(77.3)	193(81.4)		1
Trainee	58(22.7)	44(18.6)		1.3 [0.8–2.0]
**Field of activity**			0.974	
Medicine	150(58.6)	142(59.9)		1
Dentistry	41(16.0)	37(15.6)		0.9 [0.5–1.6]
Pharmacy	36(14.1)	34(14.3)		0.9 [0.4–1.8]
Paramedical	29(11.3)	24(10.1)		0.9 [0.4–1.8]
**Location of activity**			0.020	
North of Tunisia	89(34.8)	107(45.1)		1
Center of Tunisia	101(39.5)	87(36.7)		1.4 [0.9–2.1]
South of Tunisia	65(25.4)	40(16.9)		2.0 [1.2–3.2]
**Sector of activity**			0.025	
Private	69(27.0)	86(36.3)		1
Public	187(72.4)	151(63.7)		1.5 [1.1–2.3]
**Frequency of direct contact with COVID-19 inpatients**			0.141	
Every day	39(15.2)	43(18.1)		1
Sometimes	84(32.8)	92(38.8)		1.0 [0.6–1.7]
Never	133(52.0)	102(43.0)		1.4 [0.9–2.4]
**History of chronic condition**			0.666	
No	200(78.1)	178(75.1)		1
Yes	51(19.9)	50(21.1)		0.9 [0.6–1.4]
**History of allergy**			0.084	
No	212(82.8)	181(76.4)		1
Yes	39 (15.2)	50 (21.1)		0.7 (0.4–1.1]
**History of infection by the SARS-COV-2**			< 0.001	
No	255(99.6)	208 (87.8)		1
Yes	1 (0.4)	29 (12.2)		0.028 [0.004–0.2]
**Vaccination against influenza during the current season**			0.274	
No	182(71.4)	157(66.8)		1
Yes	73(28.6)	78(33.2)		0.8 [0.5–1.2]
**Sources used to be informed about the SARS-CoV-2**				
Social media			0.026	
No	74 (28.9)	89 (37.6)		1
Yes	182(71.1)	148(62.4)		1.5 [1.01–2.1]
Radio stations			0.392	
No	159 (62.1)	151 (63.7)		1
Yes	97(37.9)	86(36.3)		1.1 [0.7–1.5]
Television channels			0.096	
No	105 (41.0)	112 (47.3)		1
Yes	151(59.0)	125(52.7)		1.3 [0.9–1.8]
The national web site of the Ministry of Health			0.142	
No	159 (62.1)	135 (57.0)		1
Yes	97 (37.9)	102 (43.0)		0.8 [0.6–1.2]
The web site of the Pasteur institute of Tunis			0.064	
No	240 (93.8)	211 (89.0)		1
Yes	16(6.3)	26(11.0)		0.5 [0.3–1.04]
The Tunisian web site of the Observatory of new and emergent diseases			0.455	
No	189 (73.8)	177 (74.7)		1
Yes	67(26.2)	60(25.3)		1.1 (0.7–1.6]
The Tunisian web site for health professionals “SAUVE.tn”			0.027	
No	242 (94.5)	212 (89.5)		1
Yes	14(5.5)	25(10.5)		0.5 [0.2–0.97]
The Tunisian web site for information about COVID-19 “Covid.tn”			0.003	
No	172 (67.2)	130 (54.9)		1
Yes	84(32.8)	107(45.1)		0.6 [0.4–0.8]
Newspapers			0.161	
No	211 (82.4)	186 (78.5)		1
Yes	45(14.6)	51(21 .5)		0.8 [0.5–1.2]
Websites of international scientific organizations			0.490	
No	138 (53.9)	129 (54.4)		1
Yes	118(46.1)	108(45.6)		1.0 [0.7–1.5]
Scientific journals			0.463	
No	107 (41.8)	101 (42.6)		1
Yes	149(58.2)	136(57.4)		1.0 [0.7–1.5]
**Perceptions**				
** Lack of information about the SARS-CoV-2 vaccines**			0.008	
No	36(14.1)	54(22.8)		1
Yes	220(85.9)	183(77.2)		1.8 [1.1–2.9]
** Concerns about the components of the upcoming vaccines**			0.002	
No	66 (25.8)	90(38.0)		1
Yes	190(74.2)	147 (62.0)		1.8 [1.2–2.6]
** High or very high risk of infection by SARS-CoV-2**			0.477	
No	87(34.0)	79(33.3)		1
Yes	169(66.0)	158(66.7)		1.0 [0.7–1.4]
** High or very high risk of complications in case of infection by SARS-CoV-2**			0.061	
No	209(81.6)	179(75.5)		1
Yes	47(18.4)	58(24.5)		0.7 [0.4–1.1]

Perception of a lack of information about the SARS-CoV2 vaccination was positively associated with SARS-CoV2 vaccine hesitancy with a proportion of 85.9% among hesitant participants versus 77.2% in non-hesitant ones (*p* = 0.008). Use of social media was also positively associated with hesitancy towards the vaccination among participants (6.3% among hesitating participants versus 11% among those not; *p* = 0.043) contrary to the use of the national website of the Pasteur Institute or the national website for information about COVID-19 “Covid.tn” which were significantly associated with less hesitancy (Table 3).

Otherwise, thinking that the upcoming vaccines may contain harmful components was reported by 74.2% of hesitant professionals versus 62% among the rest of participants (*p* = 0.002). More details about the hesitancy towards the COVID-19 vaccination according to the individual characteristics of participants are displayed in Table 3.

Table 4 details the results of the binary logistic regression analysis for the factors related to SARS-CoV-2 vaccine hesitancy among participants. The magnitude of associations presented in “model 1” did not differ substantially from those in “model 2” from which the variable age was excluded. This indicate that the age is not an effect modifier. The variation of 20% between the values of the crude and the adjusted odds ratios for age may be due to a confounding effect. Similarly for “model 3” from which, the variable “Previous SARS-CoV-2 infection” was excluded to avoid an eventual convergence failure issue. Indeed, by excluding the variable “age,” the magnitude of associations did not differ substantially from those in “model 2” while working in the public sector and less frequent contact with COVID-19 patients were revealed to also predict more hesitancy among participants. On another note, the three models showed that working in the south of the country predicted the most hesitancy compared to those exercising in the central of Tunisia and with reference to those exercising in the north of the country. Concerns regarding the components of the upcoming vaccines represented another predictor of SARS-CoV-2 vaccine hesitancy with an adjusted OR comprised between 1.2 and 2.7. On the other hand, the use of the national website for information about COVID-19 (covid.tn) predicted less hesitancy with an adjusted OR of 0.6 [0.4–0.9].

**Table 4:** Binary logistic regression analysis for characteristics related to SARS-CoV-2 vaccines hesitancy among participants

10			Model 1^a^		Model 2^a^			Model 3^a^
**Variables**	***p*-value**	**Crude OR [95% CI]**	***p*-value**	**Adjusted OR [95% CI]**	***p*-value**	**Adjusted OR [95% CI]**	***p*-value**	**Adjusted OR [95% CI]**
**Age**	0.003		0.067				0.056	
≥ 40 years		1		1				1
< 40 years		1.8 [1.2–2.6]		1.5 [0.97–2.2]				1.5 [0.98–2.2]
**Sex**	0.047		0.036		0.013		0.324	
Male	-	1		1	-	1		1
Female		1.5 [1.1–2.2]		1.6 [1.03–2.5]		1.7 [1.1–2.7]		1.2 [0.8–1.9]
**Location of activity**	0.011		0.009		0.010		0.011	
North of Tunisia	-	1		1	-	1		1
Central of Tunisia		1.4 [0.9–2.1]		1.3 [0.8–2.0]		1.3 [0.8–2.0]		1.3 [0.8–2.0]
Southern of Tunisia		1.9 [1.2–3.2]		2.3 [1.3–3.9]		2.3 [1.3–3.9]		2.2 [1.3–3.7]
**Having been already infected by the SARS-CoV-2**	< 0.001		< 0.001		< 0.001			
No	-	1		1	-	1		
Yes		0.028 [0.004–0.2]		0.024 [0.003–0.2]		0.024 [0.003–0.2]		
**Use of the national site for information about COVID-19: covid.tn**	0.005		0.015		0.010		0.011	
No	-	1		1	-	1		1
Yes		0.6 [0.4–0.8]		0.6 [0.4–0.9]		0.6 [0.4–0.9]		0.6 [0.4–0.9]
**Think that the vaccines that will be available in Tunisia may contain harmful components**	0.004		0.007		0.006		0.007	
No	-	1		1	-	1		1
Yes		1.8 (1.2–2.6]		1.8 [1.2–2.7]		1.8 [1.2–2.7]		1.7 [1.2–2.6]
**Sector of activity**	0.025						0.025	
Private		1						1
Public		1.5 [1.1–2.3]						1.6 [1.1–2.5]
**Frequency of direct contact with COVID-19 inpatients**	0.141						0.035	
Every day		1						1
Sometimes		1.0 [0.6–1.7]						1.1 [0.6–1.9]
Never		1.4 [0.9–2.4]						1.8 [1.03–3.2]

^a^The following co-variables were introduced to the initial model: age, gender, location of activity, sector of activity, Frequency of direct contact with COVID-19 inpatients, History of allergy, History of infection by the SARS-CoV-2, use of Social media, use of Television channels, use of The national web site of the Ministry of Health, use of the web site of the Pasteur institute of Tunis, use of the Tunisian web site for health professionals “SAUVE.tn”, use of the Tunisian web site for information about COVID-19, use of newspapers, perception of a lack of information about the SARS-CoV2vaccines, concerns about the components of the upcoming vaccines, perception of a high or very high risk of complications in case of infection by SARS-CoV2

## Discussion

To the best of our knowledge, this is the first Tunisian study that aimed at evaluating the acceptance of SARS-CoV-2 vaccines among all the categories of health professionals working in primary, secondary and tertiary care centers. It would provide a baseline reference for future evaluations. Our results would also serve for neighboring countries and other limited income countries in planning for their vaccination strategies.

Our study highlighted that between the 7^th^ and the 21^th^ of January 2021, 66.3% of Tunisian health professionals were thinking that they have high or very high risk of SARS-CoV-2 infection while 21.3% were thinking that they risk serious complications in case of infection. Acceptance rate of SARS-CoV-2 vaccine was 35.5% (95% CI: 31.3–39.7) whereas the prevalence of SARS-CoV-2 vaccine hesitancy was 51.9% (95% CI_:_ 47.5–56.3). Working far from the capital (in the south or in the central of Tunisia) and concerns about the vaccines components predicted more hesitancy among participants. In contrast, the use of the national COVID-19 information website predicted less hesitancy among them.

Despite the high rates of risk perception, only 35.5% of healthcare professionals in our sample were readily willing to get the SARS-CoV-2 vaccine. This low rate of vaccine acceptance is far away from the herd immunity targets [6]. It joins that in USA (36%) [15] and in Qatar (33.8%) [16]. However, it is lower than those in France (76,9%) [29], Italy (67%) [14] and Greece (78.5%) [30] and higher than those in several Arab countries [16]. Among paramedical participants, this rate was of 20.8%. Low vaccination acceptance rates were found among non-physician healthcare workers in other countries [31]. Otherwise, the hesitancy rate (51.9%) revealed by the current study was higher than that reported after an online opinion survey conducted among the same target population between the 10^th^ and the 20^th^ of January 2021 (33.6%). Nonetheless, this opinion survey showed a higher refusal rate of 23.5% [32]. The used sampling method during this online opinion survey was not reported. However, repartition of participants from the different fields of activities was similar to that observed in our study [32].

As reported in previous studies [33], participants were mostly females. The trend of feminization in the Tunisian health sector may explain somewhat this female predominance [34]. Analyzing hesitancy among participants according to the sex showed that females were less willing than males to uptake the SARS-CoV-2 vaccine. This result is harmonious with several other studies [12]. The higher male acceptance of vaccine may be due to a greater propensity for risk taking [35]. This also could be related to concerns among females about higher risks of induced autoimmune diseases or fertility problems as it was spread on social media [36, 37].

Older respondents were significantly less hesitant to uptake the SARS-COV-2 vaccine. However, having a chronic condition or allergy did not seem to contribute to this hesitancy among them. A recent scoping review reported that individuals of older age are more likely to accept COVID-19 vaccines [12]. This was explained by a perception of greater vulnerability to SARS-CoV-2 infection but also by higher education and greater experience in healthcare [12]. Indeed, among our participants, those who were trainees were more likely to be hesitant than those who have graduated.

Having its professional activity far from the north of the country (where is located the capital) predicted more hesitancy among participants. In line with this result, lower vaccination rates among deprived groups were observed in several surveys [15, 38, 39]. More efforts should be provided in the Tunisian underserved regions, especially in the south, in order to overcome regional disparities in terms of vaccination against SARS-CoV-2.

Professionals from private sector were significantly less hesitant to get the SARS-CoV-2 vaccine. This joins the results of a study led in Hong Kong [40]. This may be explained by economic reasons. In fact, in private sector; sick leave in case of COVID-19 episode is not regularly paid in Tunisia.

More frequent contact with COVID-19 patients was associated with less hesitancy towards the SARS-CoV-2 vaccine among participants. Indeed, a realistic risk perception allows the implementation of voluntary preventive behaviors [33]. On another note, having been previously infected by SARS-CoV-2 predicted less hesitancy among participants. Divergent results were reported regarding the association between previous COVID-19 infection and willingness to receive a SARS-CoV-2 vaccine [41–43]. These divergent attitudes may be explained by the lack of knowledge about the duration of protective immunity after infection by this new virus [44].

Among participants, 81.7% reported lack of information about SARS-CoV-2 vaccines. Social media were the most used information source by them, which joins the results of an Egyptian study [45]. Lack of information and use of social media to be informed about SARS-CoV-2 vaccines were both significantly associated with more hesitancy towards these vaccines among participants. These results corroborate those of similar studies led in Egypt and Italia [11, 21] [14, 46]. Concerns about the vaccines components represented another predictor of SARS-CoV-2 vaccine hesitancy among participants. In fact, doubts regarding the SARS-CoV-2 vaccines safety among health professionals were reported in several countries such Italy [14], Democratic republic of Congo [39] and Egypt [45]. Differently, the use of the official national websites was significantly associated with less hesitancy rates among participants. Similar result was observed in Saudi Arabia [41]. Indeed, improved information on vaccines has been shown to increase vaccines’ acceptance [47].

Focusing on the vaccination campaign in Tunisia, we can note that although the launch of the online registration to get the vaccine since 15 January 2021 [25], the vaccination did not start before 13 March 2021. In fact, there were difficulties to obtain vaccines doses [48]. Health professionals represented the first priority group [26]. The concomitant communication plan included a first step of registration promotion during February 2021 with disinformation countering via mass media and social media. The second step began in March 2021. It aims to facilitate registration of people and to inform them about where and when they can beneficiate from the vaccine [26]. After one year of the onset of the vaccination campaign, proportion of vaccinated health professionals is still unavailable [25, 26, 49]. In addition, the incidence of COVID-19 among health professionals does not figure on the periodic national reports [25, 26, 49]. As of 7 March 2022, four reports were published (since September 2021) about the recorded side effects among the vaccinated people [50]. However, the content of these reports was not disseminated through the official website of the ministry of health [26] or the national vaccination portal [25].

SARS-CoV-2 vaccine scarcity in Tunisia and poor resources [51] should not discourage policy makers to implement an effective information campaign. Involving health professionals, especially Public Health specialists, in this campaign would increase confidence in the vaccines, as they are experts in prevention methods. Involving partners from the other sectors such as anthropologists, artists and national leaders is also recommended. Sharing updated information with health professionals during periodic sessions would encourage hesitant ones to uptake the vaccine. Especially, females, the youngest ones, paramedical professionals and those in the underserved regions. The content of these sessions should focus on the severity of COVID-19 episodes and the impact of adherence to self-protective behaviors [52]. To increase confidence in the vaccines, the broadcast messages should report the development methods and the protection mechanisms of the SARS-CoV2 vaccines. Organizing vaccination sessions in the occupational health centers would encourage these groups to uptake the vaccine. Facebook represent another way for disseminating valid messages and tackling misinformation about the vaccines, especially that it represents the most famous social media platform in Tunisia [16]. Engaging health care professionals in social media to counter the vaccines’ related misinformation would improve the vaccine acceptance among the other health professionals and the general population as well. In fact, the general population considers them trustworthy.

More solidarity at the international level is required for a global COVID-19 vaccine equity. Otherwise, we risk the emergence and the spread of new variants of the SARS-CoV-2 which could threaten vaccinated and not vaccinated people worldwide.

Results of the current study should be interpreted with taking into account some limitations. Firstly, the cross sectional nature of the study did not allow to report causal relationships but only statistical associations. Besides, random sampling was not possible as no lists of national or regional health professionals were available. However, the required sample size was reached. Moreover, although that Public Health professionals were not represented in our sample because of their reduced number in Tunisia, the main categories of the health professionals were represented. Finally, attitudes and perceptions were self-reported by participants, which might lead to a social desirability bias. Nonetheless, data were collected anonymously and participation was voluntary.

## Conclusion

An effective national information campaign is required to reassure the Tunisian health professionals regarding the safety of COVID-19 vaccines. Females, the youngest ones, paramedical professionals and those in the underserved regions deserve more attention. Social media would represent a cost effective tool for this campaign.

## Data Availability

The datasets generated and analyzed during the current study are not publicly available due to limitations of ethical approval involving the patient data and anonymity but are available from the corresponding author on reasonable request.

## Abbreviations

COVID-19: Coronavirus disease 2019
SARS-CoV-2: Severe acute respiratory syndrome coronavirus 2
